# Age-Related Alterations in the Expression of Genes and Synaptic Plasticity Associated with Nitric Oxide Signaling in the Mouse Dorsal Striatum

**DOI:** 10.1155/2015/458123

**Published:** 2015-03-04

**Authors:** Aisa N. Chepkova, Susanne Schönfeld, Olga A. Sergeeva

**Affiliations:** Department of Molecular Neurophysiology, Medical Faculty, Heinrich Heine University, 40225 Düsseldorf, Germany

## Abstract

Age-related alterations in the expression of genes and corticostriatal synaptic plasticity were studied in the dorsal striatum of mice of four age groups from young (2-3 months old) to old (18–24 months of age) animals. A significant decrease in transcripts encoding neuronal nitric oxide (NO) synthase and receptors involved in its activation (NR1 subunit of the glutamate NMDA receptor and D1 dopamine receptor) was found in the striatum of old mice using gene array and real-time RT-PCR analysis. The old striatum showed also a significantly higher number of GFAP-expressing astrocytes and an increased expression of astroglial, inflammatory, and oxidative stress markers. Field potential recordings from striatal slices revealed age-related alterations in the magnitude and dynamics of electrically induced long-term depression (LTD) and significant enhancement of electrically induced long-term potentiation in the middle-aged striatum (6-7 and 12-13 months of age). Corticostriatal NO-dependent LTD induced by pharmacological activation of group I metabotropic glutamate receptors underwent significant reduction with aging and could be restored by inhibition of cGMP hydrolysis indicating that its age-related deficit is caused by an altered NO-cGMP signaling cascade. It is suggested that age-related alterations in corticostriatal synaptic plasticity may result from functional alterations in receptor-activated signaling cascades associated with increasing neuroinflammation and a prooxidant state.

## 1. Introduction

Normal aging is associated with declining sensorimotor control and cognitive functions which may result from changes in the cortex-basal ganglia circuits involved in planning, initiation, and control of voluntary movements. Along with a gradual partial atrophy of the basal ganglia with advanced aging human brain imaging studies revealed age-related alterations in the basal ganglia-neocortex connectivity at rest and during execution of motor tasks [1–3]. Functional organization and rearrangement of networks involved in learning and execution of motor skills is thought to be associated with long-term changes in corticostriatal neurotransmission [4–6]. Two major forms of synaptic plasticity, long-term depression (LTD) and long-term potentiation (LTP) of corticostriatal neurotransmission, have been shown in the rodent striatum [4, 7]. As a primary input structure of the basal ganglia the striatum receives cortical information through topographically organized glutamatergic projections to its principal medium size spiny neurons which integrate and transfer it to the output structures under control of dopaminergic input from the substantia nigra and striatal cholinergic and nitrergic interneurons. This interaction of dopamine, acetylcholine, and nitric oxide neurotransmitter systems determines whether corticostriatal transmission is amplified (LTP) or dampened (LTD) following repetitive activation [8]. Although numerous neurochemical and pharmacological studies have reported alterations in all major striatal neurotransmitter systems with aging [9–13], only a few analysed alterations in corticostriatal synaptic plasticity in animal models of normal aging showing an age-related decrease in short-term plasticity [14] and some deficit in two different forms of long-term plasticity associated with activation of N-methyl-D-aspartate- (NMDA-) type glutamate receptors (NMDAR) [14, 15].

One of the key modulators of striatal neuronal activity is nitric oxide (NO) whose production by striatal nitrergic interneurons is stimulated by activation of glutamatergic corticostriatal and dopaminergic nigrostriatal pathways through NMDAR and D1-like dopamine receptors (D1R) [16]. NO regulates, through its physiological receptor soluble guanylate cyclase (sGC), producing cyclic guanosyl monophosphate (cGMP), short- and long-term plasticity at corticostriatal synapses in medium spiny neurons [17–20]. Aging is associated with considerable reduction in the number of striatal neurons containing NO synthase [21, 22] suggesting a significant decrease in NO production and corresponding alterations in NO-dependent processes. In fact, the data on age-related changes in the striatal NO synthase (NOS) activity and in NO-cGMP-protein kinase G (PKG) signaling are controversial [23–25]. The aim of the present study was to investigate age-related alterations in the expression of genes involved in NO signaling and to explore the manifestation of several forms of NO-dependent plasticity in the dorsal striatum of mice at four different ages. We found that striatal tissue from old (≥18 months) mice is characterized by decreased expression of major genes involved in NO production, namely, genes encoding for the essential NR1 subunit of the NMDAR, D1R, and neuronal NOS (nNOS). Analysis of NO-dependent plasticity of corticostriatal neurotransmission revealed that aging is associated with alterations in the expression of electrically induced LTP and LTD and with a significant decrease in long-term depression of responsiveness after pharmacological activation of group I metabotropic glutamate receptors (group I mGluR) with (S)-3,5-dihydroxyphenylglycine (DHPG-LTD). Pharmacological inhibition of cGMP degradation recovered DHPG-LTD suggesting the impaired NO-cGMP signaling as a cause of its age-related deficit.

## 2. Materials and Methods

### 2.1. Animals

Male GFP-GFAP transgenic mice at the age from 2 to 24 months were used. Green fluorescent protein (GFP) integrated in the mouse genome under control of the GFAP promoter has the ability to fluoresce when irradiated by ultraviolet light and its simultaneous expression with GFAP allows the visualization of astrocytes in the mouse brain.

Transgenic mice FVB/N-Tg(GFAPGFP)14 Mes/J (details on genotype can be found in [26]) purchased from Jackson Laboratories (Stock # 003257, Jaxmice, US) were bred and aged in our facility. Male mice were kept in groups (2–6 animals per cage) on a 12 h day-12 h night light schedule with* ad libitum* access to food and water. Four age groups were selected for the analysis: group I (2-3 months old), group II (6-7 months old), group III (12-13 months old), and group IV (18–24 months old). All procedures were in compliance with German law and were approved by the University of Dusseldorf authorities.

### 2.2. Striatal Slices Preparation

**Figure 1:**
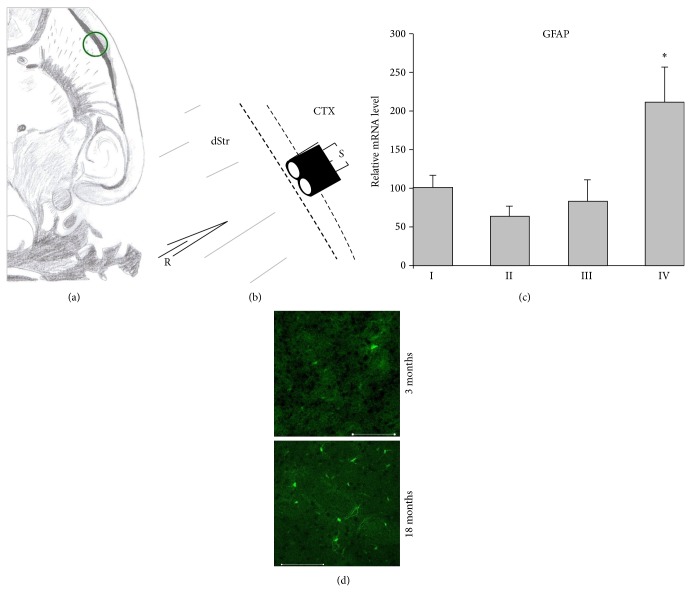
Increased expression of GFAP in the dorsal striatum of old (group IV) mice. (a) Schematic diagram of a horizontal brain slice used for the histological and electrophysiological analysis. The region of counting the GFAP-expressing glial cells and field recording is marked by the green circle. (b) Schematic drawing of relative position of stimulating (S) and recording (R) electrodes in the region marked by the green circle in (a) dStr: dorsal striatum, CTX: cortex. (c) Relative levels of striatal GFAP mRNA in different age groups: group I (*n* = 8), group II (*n* = 7), group III (*n* = 4), and group IV (*n* = 4). (d) Examples of confocal microscope images with eGFP-GFAP fluorescent cells in striatal slices from a young and an old mouse. Scale bars: 100 *µ*m.

Horizontal brain slices containing striatum and hippocampus (400 *μ*m, Figure 1(a)) were prepared with a vibratome (Campden Instruments, UK) from the brain immersed in an ice-cold modified artificial cerebrospinal fluid (aCSF) with complete sucrose substitution for NaCl and collected in a glass dish filled with the same solution. Corticostriatal preparations were dissected from the horizontal slices. One half of them were immediately treated for further histology and gene expression analysis while another half was transferred to a store glass with a standard aCSF containing (in mM) 125 NaCl, 1.8 KCl, 1.2 KH_2_PO_4_, 2.4 CaCl_2_, 1.2 MgCl_2_, 26 NaHCO_3_, and 10 D-glucose, constantly saturated with 95% O_2_/5% CO_2_ gas mixture (pH 7.4) and after at least 2 h preincubation at room temperature was used for electrophysiological experiments.

### 2.3. Histology and GFP-Expressing Cell Counting

Corticostriatal slices were fixed immediately after cutting to maximally preserve fluorescence [27]. After fixation and cryopreservation (1 h in 20% sucrose) slices were cryosectioned at 25 *µ*m thickness, mounted on gelatin-coated slides, dried, and covered with aqua poly/mount (Polysciences Inc., Warrington PA, USA). The middle portion of each section (the analyzed area seen in Figure 1(a)) was photographed on conventional fluorescence or confocal microscopes (LSM META 510, Zeiss, Germany). Fluorescent GFP-expressing cells were counted from photographs within 300 *µ*m^2^ square fields, if their soma was in focus and the intensity of fluorescence clearly exceeded the background level. Cell counts were obtained by averaging the counts from 4–10 sections (1-2 randomly selected fields per section) taken from each animal. Between-group comparison was done with Mann-Whitney *U* test.

### 2.4. PCR Array and Conventional Real-Time RT-PCR

Striatal tissue was isolated from 1–3 slices and total cellular mRNA was extracted using an mRNA isolation kit (Quickprep Micro mRNA Purification Kit, GE Healthcare, GB). The PCR array “Mouse Nitric Oxide Signaling Pathway” (PAMM-062Z, SA Bioscience) was used to detect age-related alterations in gene expression. In this array 84 wells are filled with PCR primers for the genes involved in NO signaling, oxidative stress, and antioxidant defence (see Supplementary Table 1 in Supplementary Material available online at http://dx.doi.org/10.1155/2015/458123, for the whole list of the genes) (A1-G12 samples). Seven wells are used to test nontranscribed genomic DNA contamination and PCR performance (H06–H12) and 5 wells contain primers for housekeeping genes (H01–H05): beta-actin, Actb; beta-2-microglobulin, B2m; glyceraldehyde-3-phosphate dehydrogenase, Gapdh; heat-shock protein 90, Hsp90; and glucuronidase beta, Gusb. To determine the most appropriate reference gene among them we used criteria described previously [28, 29]. As Actb showed the smallest difference between young (3 months) and old groups (18 months) and the highest *P* value in Student's *t*-test applied to individual 2^−Ct^ values, all real-time PCR reactions were normalized to Actb. One gene array was run without cDNA and served as negative control to determine the primer melting temperature (Tm). Amplified gene products consistently showing primer artefacts in their DNA melting curve, a Tm lower than 75°C, or double peaks in the DNA denaturation curve were excluded from the list of analyzed genes. Among those were the following: Epx, Gpx2, Gpx5, Hmgb1, Idh1, Noxa1, and Txnrd2 (for the full names see Supplementary Table 1).

The genes whose expression was significantly age-dependent according to the PCR array were further explored with conventional real-time RT-PCR. Additionally, we analyzed the expression of genes encoding dopamine receptors, group I mGluRs, the endocannabinoid receptor CB1, involved in corticostriatal plasticity, as well as the expression of genes associated with redox and immune status (see Supplementary Table 2).

For the reverse transcription 8 *µ*L of purified mRNA was added to 7 *µ*L of the first strand cDNA synthesis kit (GE Healthcare, GB) reaction mix. After incubation for 1 hour at 37°C the reverse transcription was stopped by freezing at −20°C. The RT-PCR was performed in an Applied Biosystems StepOne real-time PCR machine using the SYBR Green Master mix kit (Applied Biosystems). Reactions were performed in MicroAmp optical 96-well plates in a total volume of 10 *µ*L comprising the final concentration of SYBR Green PCR Master mix, cDNA (100–150 ng), and primers. The following thermal protocol was used: initial incubation at 50°C for 2 min to activate uracil N-glycosylase, 10 min at 95°C to inactivate the uracil N-glycosylase and activate the AmpliTaq Gold Polymerase, and finally 40 cycles of 15 s at 95°C, 2 min at 50°C, and 1 min at 60°C. For the conventional real-time RT-PCR 10 to 50 pM forward and reverse primers (for sequences see Supplementary Table 2) were used. Representative conventional RT-PCR products were purified in water and sequenced (known sequences to which they showed 100% identity with GENBANK are given in Supplementary Table 2). All reactions were carried out in duplicate. Standard curves for real-time PCR protocols were obtained for each primer pair. Values for *r* ^2^ (linear regression coefficient) and *E* (efficiency) were found suitable for the relative quantification of gene expression and are provided in Supplementary Table 2. Real-time PCR efficiencies were calculated according to *E* = 10^[−1/slope]^ [30]. Relative mRNA level encoding each gene was estimated by the “2^−ΔΔCt^” method as described previously [27], where ΔCt = Ct target gene- Ct Actb. Average 2^−ΔΔCt^ values for the young group were taken as 100% and the individual values for other age groups were expressed as percentage of this value.

### 2.5. Field Potential Recording and Data Analysis

After at least 2 h preincubation at room temperature a single slice was transferred to a submersion-type recording chamber where it was continuously perfused with a standard aCSF at a flow rate of 1.5–2 mL/min at 32°C.

A bipolar nickel-chrome stimulation electrode was positioned on the subcortical white matter at the border between cortex and striatum (Figure 1(b)). Corticostriatal field potentials were recorded with low-resistance (2–4 mOm) aCSF-filled micropipettes positioned within the striatum at a distance up to 0.3 mm from the stimulation electrode. After the initial testing of stimulus-response relationships, the stimulus intensity was adjusted to induce a postsynaptic peak response of about 50%–60% of its maximal value and stimulation frequency was set to 0.033 Hz. Each experiment included a 15–20 min period of control recording, application of high-frequency electrical stimulation (HFS) or chemical stimulus, and 60-min monitoring of poststimulus alterations in responsiveness. HFS consisted of 4 trains of 100 stimuli at 100 Hz with 10 sec intertrain intervals. The chemical stimuli were 10-min perfusion with 100 *µ*M S- 3,5,-dihydroxyphenylglycine (DHPG) or 10–15 min perfusion with phosphodiesterase inhibitor, zaprinast or rolipram. DHPG was purchased from Abcam (Germany) and zaprinast and rolipram were purchased from Sigma. The drugs were prepared as stock solutions, stored in aliquots at −20°C, and defrozen and diluted by aCSF before application.

Signals were amplified, digitized at 10 kHz, stored on a hard disk of a PC using Clampex software of pClamp (Axon Instruments), and analyzed offline, using Clampfit and Excel software. Ten consecutive field responses (5-min recordings) were averaged. The averaged postsynaptic peak response was measured as an average of its minimum (from the early positive peak to the peak negativity) and maximum (from the peak negativity to the late positivity peak) values. All measurements were normalized to baseline (the mean value for a 15–20 min period before conditioning stimulus) and plotted against time. The presence of long-term alterations of corticostriatal neurotransmission after HFS was determined by a persistent deflection of the peak amplitude by ±15% from baseline, and the magnitude of LTD or LTP was calculated from the data points acquired during the last 20 min of recording. Changes in responsiveness induced by chemical stimuli were evaluated by comparing the response values averaged through the first and the last 20-min periods of poststimulus recordings with the baseline.

### 2.6. Statistical Analysis

The data were statistically analysed using GraphPad Prism 5 software and are presented as mean ± SEM with *n* indicating the number of slices per group (each group included slices from at least 4 animals). Gene expression data were analysed using one-way analysis of variance (ANOVA) with the Dunnett's posttest and Mann-Whitney *U* test. Between-group comparisons of electrophysiological data were performed using one-way and two-way ANOVA with the Dunnett and Bonferroni posttests. Paired *t*-test and Wilcoxon matched-pair signed rank test were used for evaluating drug effects. Chi-square test and Fisher's exact probability tests were used for the analysis of LTD/LTP distribution.

## 3. Results

### 3.1. Astrogliosis and Expression of Aging Markers

Old age is associated with astrogliosis, low-grade inflammation, and oxidative stress in the brain [31–33]. As the life span of FVB mice, the genetic background for generation of GFP-GFAP mice, varies within 1.5–2 years [34], transgenic mice at the age of 18 months may be classified as old animals. To substantiate this classification we compared the expression of glial, oxidative stress and inflammation markers in striatal tissue of 2-3-month-old (group I) and 18–20-month-old mice (group IV), referred to as young and old mice.

Semiquantitative RT-PCR analysis revealed significant upregulation of the striatal level of GFAP mRNA in old mice (Figure 1(c)). According to RT-PCR analysis the striatum of old mice showed also significantly higher transcript levels for some other glial markers. Thus the level of the mRNA encoding for the S100*β* in old striatum (134 ± 10%, *n* = 12) exceeded significantly (*P* < 0.01) the young value (100 ± 8%, *n* = 9). Transcripts for the macrophage/microglia-specific calcium-binding protein IBA1 were also detected at a significantly higher level in old mice (123 ± 7%, *n* = 10 versus 100 ± 7%, *n* = 10 in the young, *P* < 0.05). Levels of transcripts encoding for the glutamate transporter 1 (GLT-1), glutamine synthetase (GS), and oligodendrocyte marker myelin-associated glycoprotein (MAG) did not differ between old and young striatal samples. The counting of GFP-fluorescent cells showed that the number of GFAP-expressing cells in the striatum of old mice exceeds the number in young mice about threefold (7.8 ± 0.4 per field, 6 mice, versus 2.7 ± 0.7 per field, 7 mice, *P* < 0.005, Mann-Whitney *U* test (Figure 1(d)).

In addition to the increased expression of major glial markers, striatal tissue from old mice showed significantly increased levels of transcripts encoding oxidative stress and immunity (Table 1). It is noteworthy that an increase in beta-2-microglobulin (B2m) and lipocalin-2 (Lcn2) attained the level of significance already in the striatum of middle-aged, 12-13-month-old mice.

### 3.2. Expression of Receptors and Enzymes Involved in Nitric Oxide Signaling

**Figure 2:**
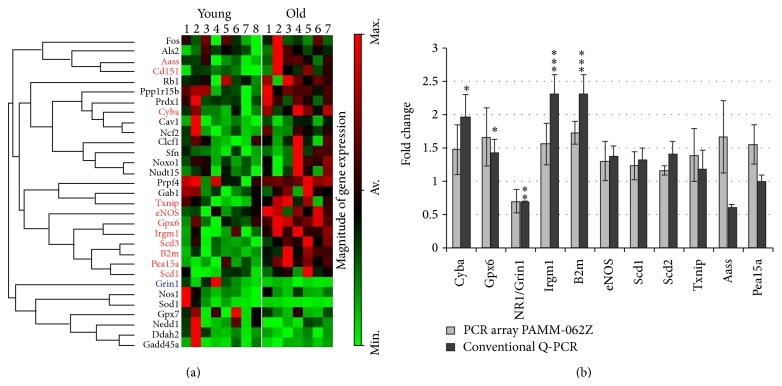
Gene array analysis of striatal transcriptome in young and old mice. (a) Cluster diagram analysis of 31 selected genes expressed in young (*n* = 8) and old (*n* = 7) mice. Separation of all samples into 2 age groups was automatically done by the analysis program for only these 31 out of 84 genes included in the PAMM-062Z array. The groups of genes connected by lines on the left of the cluster diagram can be considered coregulated genes. Names of genes whose expression significantly increased with age are written in red. Note an increased expression of genes known to be involved in the immune response (B2m, Irgm1) and oxidative stress (Scd1, Txnip, Gpx6, and Cyba). (b) Validation of gene array data with conventional RT-PCR (primers are given in Supplementary Table 2). Note that significant alterations in the relative mRNA levels for eNOS, Txnip, Aass, and Pea15a revealed by gene array were not confirmed by RT-PCR, whereas transcriptional downregulation with aging for the NMDA receptor NR1 subunit (Grin1) was detected by both assays.

Comparative RT-PCR analysis of mRNAs encoding the receptors and enzymes involved in regulation of corticostriatal synaptic plasticity through NO signaling revealed a tendency to lower expression of transcripts for all subunits of NMDAR with aging; however this decrease reached the significant level only for the NR1 (Grin1) subunit (Figure 2). The striatum of old mice showed also a significantly reduced expression of D1R and nNOS (Table 1). In contrast to nNOS, the level of mRNA encoding the endothelial NOS (eNOS) tended to be increased with advanced age (Table 1). Thus, old striatum was characterized by opposite changes in the expression of mRNA encoding the two major NO-synthetising enzymes, nNOS and eNOS, both of which are involved in regulation of corticostriatal synaptic plasticity [17, 18, 20] and by the diminished expression of mRNA encoding NMDAR and D1R, the principal receptors activating NO synthesis by the striatal nitrergic interneurons [16].

### 3.3. Age-Dependent Alterations in Corticostriatal Neuroplasticity

In the rodent dorsal striatum, two forms of plasticity at excitatory corticostriatal synapses are strictly NO-dependent: LTD induced by the high-frequency stimulation (HFS) of the cortical input, HFS-LTD [17], and LTD induced by the pharmacological activation of group I mGluRs with the specific agonist DHPG, DHPG-LTD [20]. HFS-induced corticostriatal LTP in mice also partially depends on NO synthesis [18]. We compared the expression of these forms of corticostriatal plasticity in striatal slices prepared from the brains of mice at different ages.

#### 3.3.1. Alterations in the Long-Term Effects of Electrical Stimulation of the Corticostriatal Pathway

**Figure 3:**
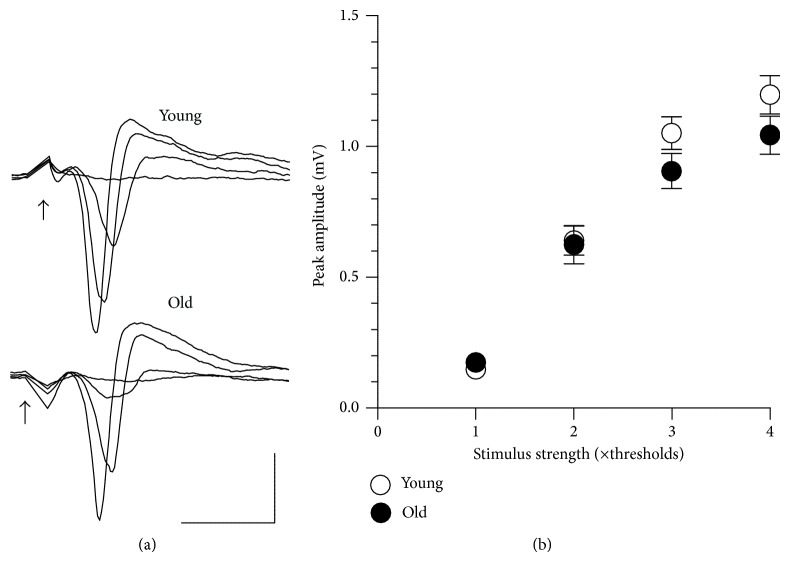
Basal corticostriatal neurotransmission undergoes no significant age-related alterations. (a) Representative examples of corticostriatal field responses recorded in slices from young adult (group I) and old (group IV) mice at *n*-fold increasing stimulus strength. Each trace represents an average of three responses to the same stimulus voltage. Stimuli are indicated by arrows. Calibrations: vertical: 0.5 mV, horizontal: 5 msec. (b) Averaged stimulus-response plots obtained from corticostriatal preparations from group I (open circles, young, *n* = 15) and group IV (filled circles, old, *n* = 18) mice.

The corticostriatal field response includes a high-amplitude negative peak reflecting a population-spike discharge of principal striatal cells [35, 36]. To assess the properties of basal neurotransmission we analysed the stimulus-response relationships in the striatal slices from animals at different ages. Although the preparations from old animals showed slightly lower responsiveness at high stimulus intensities (Figures 3(a) and 3(b)), the two-way ANOVA using age and stimulus intensity as the between- and within-group factors, respectively, revealed no significant age-related differences in stimulus-response relationships between the four studied age groups.

**Figure 4:**
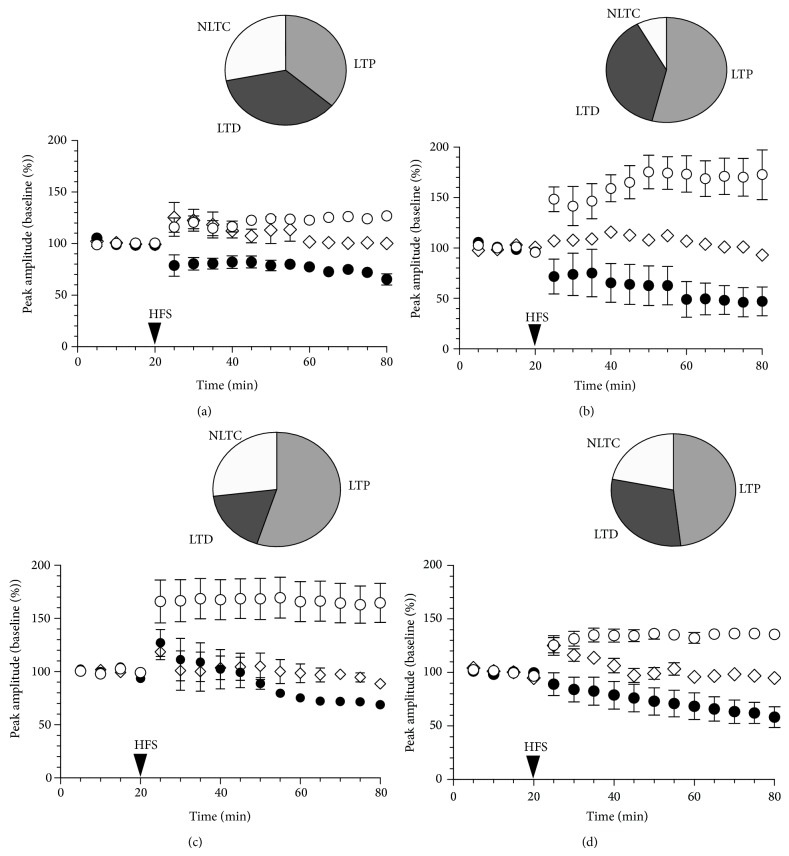
Long-term alterations in corticostriatal neurotransmission after high-frequency stimulation of synaptic input in mice of four age groups. The time course (plots) and the incidence (circle diagrams) of long-term potentiation (LTP, open circles), long-term depression (LTD, filled circles), and no long-term changes (NLTC, diamonds) in the striatum of group I (a), group II (b), group III (c), and group IV (d) mice.

In the striatal slices from young mice (group I), HFS delivery to the cortical input produced LTD, LTP, or no long-term changes (NLTC) of corticostriatal field responses with approximately equal probability (Figure 4, Table 2). Analysis of distribution of HFS outcome in four age groups did not reveal any significant difference between them (Chi-square test, *P* > 0.05). Comparative analysis of LTD and LTP magnitudes showed that both LTD and LTP were expressed at the highest levels in group II mice (Figure 4, Table 2). The slices from aged mice (groups III and IV) displayed a difference from younger slices in the time course of LTD: in the striatum of group I and II mice HFS-induced depression manifested itself within several minutes after the conditioning stimulus and persisted at a relatively constant level to the end of the 60-min observation period (Figures 4(a) and 4(b)), whereas the comparable level of depression in the slices from older mice arose only gradually (Figures 4(c) and 4(d)), and LTD was often preceded by a transient potentiation, which seemingly impeded the manifestation of LTD.

Unexpectedly, striatal slices from group II and III mice showed considerably enhanced LTP as follows: a significantly increased LTP magnitude was accompanied by a noticeable, though not statistically significant, increase in its occurrence. In the slices from old mice (group IV), the LTP magnitude and occurrence decreased compared to middle-aged groups (Figure 4, Table 2), but still remained at a level higher than in the young striatum. Thus, normal aging was accompanied by alterations in the expression and dynamics of electrically induced corticostriatal synaptic plasticity with only insignificant shifts in the LTD/LTP balance.

#### 3.3.2. Age-Related Alterations in the Expression of DHPG-LTD

**Figure 5:**
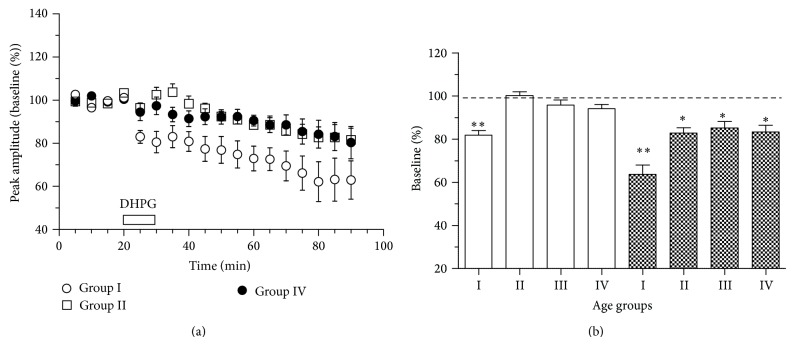
Aging weakens depression of corticostriatal responses induced by group I mGluR activation. (a) The mean time course of alterations in corticostriatal field responses induced by the group I mGluR agonist DHPG (100 *µ*M) in striatal slices from mice of different ages (*n* = 8-9 per group). (b) The magnitudes of initial (averaged through minutes 5–20 after DHPG, open bars) and long-term (averaged through minutes 55–70 after DHPG, filled bars) depression in the striatal slices from mice of four age groups. Significant decrease of corticostriatal responses compared to the baseline is marked by asterisks: ^*^ *P* < 0.05, ^**^ *P* < 0.01, paired *t*-test.

Bath application of 100 *µ*M DHPG to the striatal slices from young mice (*n* = 8) reduced corticostriatal field responses to 82 ± 2% by the end of a 10-min perfusion and this depression became even more pronounced towards the end of the observation period (64 ± 4% of baseline at 45–60 min after DHPG) (Figures 5(a) and 5(b)). In the three other age groups DHPG evoked no initial depression and much less pronounced LTD than in young slices (Figures 5(a) and 5(b)). The magnitude of DHPG-LTD in groups II (*n* = 8), III (*n* = 9), and IV (*n* = 8) constituted 83 ± 2%, 85 ± 3%, and 83 ± 3% of baseline, respectively (*P* < 0.05 with baseline, *P* < 0.001 with the young value). Thus, both the initial depression and DHPG-LTD underwent robust reduction already at 6-7 months of age and were not significantly modified with further aging.

#### 3.3.3. Age-Related Alterations in the Responses to PDE Inhibitors

Age-related variability in the expression of DHPG-LTD and electrically induced plasticity may be caused by functional alterations in intracellular signaling cascades. Since DHPG-LTD in young striatum critically depends on NO and cGMP synthesis [20], its impairment may be caused by some functional alterations in the NO-sCG-PKG signaling cascade. In turn, functional alterations in D1R-adenylate cyclase-cAMP-protein kinase A (PKA) signaling may underlie alterations in HFS-LTP. In the next set of experiments we used specific phosphodiesterase inhibitors to assess the age-related differences in the effects of intracellular accumulation of cGMP and cAMP on corticostriatal neurotransmission.

**Figure 6:**
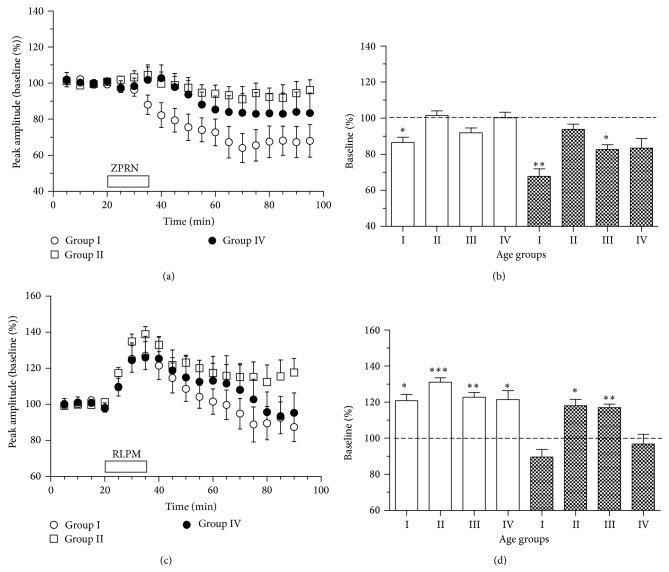
Age-dependence of alterations in corticostriatal field responses induced by phosphodiesterase (PDE) inhibitors. (a) The mean time course of alterations in corticostriatal field responses in striatal slices from mice of different ages after exposure to the inhibitor of cGMP-specific PDE5 zaprinast (ZPRN, 40 *µ*M, application period is marked by open bar). (b) Initial (averaged through minutes 10–25 after ZPRN, open bars) and long-term (averaged through minutes 60–75 after ZPRN, filled bars) changes in field response amplitudes induced by ZPRN in four age groups (*n* = 8 each). (c) The mean time course of alterations in corticostriatal field responses in striatal slices from mice of different ages after exposure to the inhibitor of cAMP-specific PDE4 rolipram (RLPM, 20 *µ*M, marked by bar). (d) Initial (averaged through minutes 5–20 after RLPM, open bars) and long-lasting (averaged through minutes 55–70 after RLPM, filled bars) changes in field response amplitudes induced by RLPM in four age groups (*n* = 7–9 per group). In (b) and (d), asterisks indicate significant decline of average response amplitude from the baseline, ^*^ *P* < 0.05, ^**^ *P* < 0.01, and ^***^ *P* < 0.001, paired *t*-test.

Short perfusion of striatal slices with zaprinast (ZPRN, 40 *µ*M), an inhibitor of cGMP-degrading phosphodiesterase PDE5, induced pronounced LTD of corticostriatal responses in the striatal slices from young mice (68 ± 4% of baseline, *n* = 8) at 60–75 min after ZPRN, whereas in the slices from older animals ZPRN evoked either no (94 ± 3% and 83 ± 5% in the striatum from group II and IV animals, *n* = 8 each) or only minor (83 ± 3% in group III, *n* = 8) depression (Figures 6(a) and 6(b)). The striatal slices from young and older animals differed also in the initial responses to ZPRN: in the young striatum significant depression of field responses was observed already during ZPRN application, whereas no significant changes in responses during this period occurred in the striatal slices from older animals (Figures 6(a) and 6(b)). Thus, the immediate and long-lasting effects of ZPRN demonstrated similar age-dependence as the effects of DHPG favouring the idea on a causal link between functional alterations in the NO-signaling cascade and DHPG-LTD suppression with aging.

Rolipram (RLPM, 30 *µ*M), an inhibitor of cAMP-specific phosphodiesterase PDE4, induced considerable (by 20–30%) enhancement of field responses in the striatal slices of all age groups (Figures 6(c) and 6(d)). In the striatum of group II and III mice (*n* = 7 each) this enhancement was persistent, so that a small but significant increase in responses was observed towards the end of monitoring (118 ± 4% and 117 ± 2% of baseline, *P* < 0.05, in groups II and III, resp.), while in the slices from the young (*n* = 9) and old (*n* = 8) groups the responses returned to the baseline after washing out the drug (90 ± 4% and 97 ± 5%, resp.). As an enhanced PKA stimulation by cAMP might determine the increased amplitude of corticostriatal LTP [7], the revealed age-related difference in the effects of elevated cAMP may underlie the significant enhancement of the HFS-LTP in the middle-aged striatum.

#### 3.3.4. Enhancement of cGMP Signaling Normalizes DHPG-LTD

**Figure 7:**
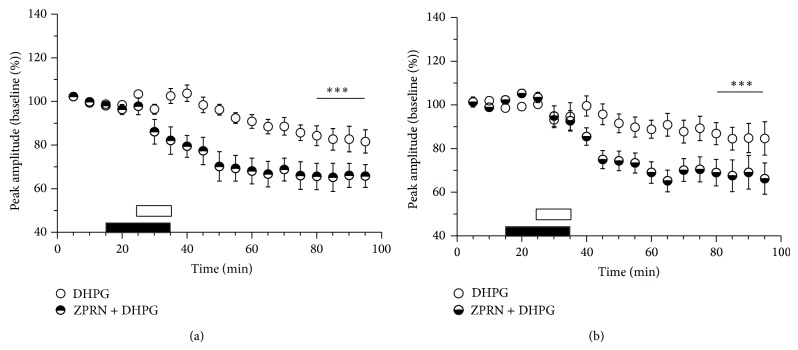
Age-related reduction of DHPG-LTD is reversed by the phosphodiesterase 5 inhibitor zaprinast (ZPRN). The time course of relative changes in field response amplitudes in corticostriatal slices from group II (a) and group III (b) mice after application of DHPG in the absence (DHPG) or presence of zaprinast (ZPRN + DHPG). Period of DHPG application is marked by open bar; ZPRN was applied 10 min before and together with DHPG (marked by filled bar). Significant differences between the DHPG-LTD magnitudes in the absence and presence of ZPRN are marked by asterisks, ^***^ *P* < 0.001, *t*-test.

Studies on neurotoxic and transgenic rodent models of Parkinson's disease showed that inhibition of cGMP hydrolysis by the PDE5 inhibitor zaprinast is able to rescue corticostriatal HFS-LTD [37, 38]. In our previous study we also found that treatment with zaprinast restored both HFS-LTD and DHPG-LTD impaired by long exposure of corticostriatal slices to hyperammonemic conditions [39]. To test whether this inhibitor could reverse age-dependent reduction of DHPG-LTD we compared the effects of DHPG application in the absence and presence of zaprinast in slices from group II and group III mice. In both groups preapplication of zaprinast increased DHPG-LTD to the level characteristic of young animals (to 66 ± 3% and 64 ± 4% of baseline in group II (*n* = 8) and III (*n* = 9), resp.). The difference with the corresponding values of control DHPG-LTD for both groups is significant at *P* < 0.001 (Figure 7).

## 4. Discussion

This study shows that advanced aging gradually increases the expression of genes regulating immune and redox states, decreases the expression of genes involved in NO-dependent synaptic plasticity (NMDAR, D1R, nNOS), and modifies the expression of some forms of corticostriatal plasticity associated with activation of NMDAR and group I mGluRs. The following age-dependent alterations in corticostriatal plasticity were observed: (i) suppression of LTD induced by pharmacological activation of group I mGluRs, DHPG-LTD; (ii) slowdown of LTD induced by electrical stimulation of the cortical input, HFS-LTD; (iii) enhanced expression of electrically induced LTP (HFS-LTP). Functional alterations in signaling cascades are considered a possible cause of age-related alterations in corticostriatal plasticity.

### 4.1. Long-Term Depression Associated with Activation of Group 1 mGluRs

At corticostriatal synapses, induction of HFS-LTD is initiated by calcium entry via L-type voltage-dependent calcium channels, coactivation of D2-like dopamine receptors (D2R), and group I mGluR-stimulating postsynaptic synthesis of endocannabinoids (eCB). It is expressed presynaptically due to retrograde signaling via activation of eCB receptors (CB1R) on cortical terminals resulting in persistent inhibition of glutamate release [4, 5, 40–42]. Induction of HFS-LTD critically depends also on activation of NO-cGMP signaling pathway [17]. Chemical LTD which is evoked in the dorsal striatum by brief activation of group I mGluRs with the specific agonist DHPG (DHPG-LTD) shares with HFS-LTD the major requirements such as the necessity of group I mGluRs and CB1R activation as well as dependence on the activity of NOS and NO-dependent soluble guanylate cyclase [20]. These two kinds of corticostriatal LTD displayed, however, different age-dependence: while DHPG-LTD was significantly reduced already at the age of 6-7 months remaining at the same low level with further aging, HFS-LTD magnitude peaked at this age showing largely dynamic rather than magnitude alterations in older animals.

The absence of any significant age-dependent changes in the mRNA expression for group I mGluRs, D2R, and CB1R implies that age-related alterations take place downstream of the receptors. Induction of corticostriatal LTD by DHPG requires NO-dependent synthesis of cGMP [20]; therefore a significantly decreased activity of nNOS and/or lower efficiency of the NO-cGMP-PKG cascade would explain its age-related suppression. This suggestion is supported by the parallel age-related decreases in nNOS mRNA expression, in the magnitude of DHPG-LTD, and in the responses to zaprinast, as well as by the restoration of normal DHPG-LTD by inhibition of cGMP hydrolysis with zaprinast. However, dysfunction of the NO-cGMP-PKG cascade can hardly explain age-related dynamics of corticostriatal HFS-LTD showing no amplitude reduction with aging. It should be noted that the NO-cGMP-PKG cascades associated with HFS-LTD and DHPG-LTD have different localizations: the HFS-LTD induction requires postsynaptic NO-dependent stimulation of PKG [17], while the NO-stimulated cascade in DHPG-LTD is activated downstream of CB1R, that is, located presynaptically [20]. The different age-dependence of DHPG-LTD and HFS-LTD may therefore be attributed to different age-sensitivity of pre- and postsynaptic NO-dependent regulatory mechanisms. Besides, in contrast to DHPG-LTD which is independent of D2R activation [5], HFS-LTD critically depends on the activation of both D2R and D1R [4, 43] which trigger opposite effects on the intracellular level of cAMP and consequently on PKA activity. The major PKA substrate in striatal spiny neurons is the dopamine and the cAMP-regulated phosphoprotein 32 kDa (DARPP-32), which is also the substrate for PKG [44]. Being a key regulator of protein kinase and phosphatase signaling in striatal principal neurons DARPP-32 is critically involved in responses to physiological and pharmacological stimuli and regulation of bidirectional corticostriatal plasticity [43, 45]. Enhanced magnitude of HFS-LTD in the 6-7-month-old striatum as well as slow development of HFS-LTD impeded by preceding potentiation in the striatum of older animals may be the consequences of age-related alterations in DARPP-32 activity regulation via dopamine and glutamate receptors. Age-related alterations in the cAMP-PKA function are suggested by our data on the increased sensitivity of corticostriatal neurotransmission to the inhibitor of cAMP degradation rolipram in the two middle age groups.

### 4.2. NMDAR-Dependent Synaptic Plasticity

Activation of NMDAR and D1R is required for LTP induction by HFS at corticostriatal synapses [4]. As aging is accompanied by a significant decrease in mRNA encoding the essential NR1 subunit of these receptors (our study), a decrease in the number [36, 46–48] and function [49] of striatal NMDAR, some impairment in NMDAR-associated plasticity would be expected. In fact, one previous study reported the reduced occurrence of HFS-induced LTP of corticostriatal responses in old rats [14]. Exploring corticostriatal plasticity in four age groups in mice we found more complex age-related alterations in the characteristics of HFS-LTP with its enhancement in the middle ages followed by some decline in the old age. It is of interest that the magnitude of HFS-LTP in the old striatum exceeded that in the young one. Potentiation of NMDA-evoked responses in medium spiny neurons through the D1R-cAMP-PKA pathway is thought to play a pivotal role in the induction of corticostriatal HFS-LTP [50]. In fact, an increase in intracellular cAMP was shown to promote LTP induction [51], while PKA inhibitors prevent it [4]. If D1R-dependent stimulation of postsynaptic PKA activity determines the magnitude of LTP, the enhancement of corticostriatal LTP may be attributed to the enhanced D1R-activated signaling compensating the age-related decrease in the amount of D1R mRNA (our study) and receptor number [52–55]. This suggestion is supported by our data on the distinct, long-term potentiating effects of rolipram on corticostriatal responses in the striatum of animals of 6-7 and 12-13 months old.

### 4.3. Age-Related Prooxidant and Proinflammatory State and Corticostriatal Plasticity

Oxidative damage of critical biological molecules resulted from alteration in a cellular redox state and accumulation of reactive oxygen species (ROS) is considered one of the causal factors of aging [56–58]. Our study revealed considerable upregulation of genes associated with ROS production (Cyba), antioxidant defence system (Gpx6), regulation of redox state (Txnip), and membrane lipid sensitivity to oxidation (Scd2) in the old striatum, which is consistent with previous findings [29, 59–62]. The Cyba or p22phox gene encodes the *α*-subunit of the membrane-associated NAD(P)H which is the second most important intracellular source of ROS after mitochondria [63]. Crosstalk signaling between these two sources may amplify and sustain ROS concentration [64, 65]. Upregulation of NAD(P)H oxidases has been reported for a variety of age-related diseases including neurodegenerative disorders [66–69]. The functional role of Gpx6, a member of the glutathione peroxidase family, remains poorly understood, but significant age-related upregulation of the Gpx6 gene has been recently reported for the rat cochlear nucleus [29, 61]. In the old striatum we observed a noticeable increase in the expression of Scd2 encoding stearoyl-coenzyme A desaturase, a rate-limiting enzyme in the biosynthesis of monounsaturated fatty acids. An increased content of oxidation-sensitive monounsaturated lipids in cell membranes at old age may be the cause of an age-associated increase in membrane lipid peroxidation [59]. Thioredoxin-interacting protein (Txnip) is a major intracellular regulator of redox state and inflammatory activation; therefore oxidative stress is closely associated with neuroinflammatory processes. Indeed, our study showed a gradual age-dependent increase in the expression of genes associated with the innate immunity and immune response (Irgm1, B2m, and Lcn2). Some of the immune response markers were noticeably elevated already in 6-7-month-old mice and significantly increased with further aging. One of these markers called beta-2-microglobulin (B2m) is a component of the major histocompatibility complex (MHC) class I molecules, which is necessary for MHC expression on the cell surface. Another marker, lipocalin-2 (Lcn2), is an innate immune protein which is synthesized and secreted from activated microglia, reactive astrocytes, neurons, and endothelial cells and is considered to be a major actor in orchestrating neuroinflammatory processes [70].

Despite the increased expression of prooxidant and proinflammatory state markers and reduced expression of plasticity-associated genes, old mouse striatum did not show such dramatic alterations in the expression of HFS-induced corticostriatal synaptic plasticity as the loss of HFS-LTD and overexpression of HFS-LTP reported for the 3-nitropropionic and 6-hydroxydopamine models of neurodegenerative diseases associated with oxidative stress and neuroinflammation [37, 71–73]. At the same time, the suppression of DHPG-LTD and the enhancement of HFS-LTP with advanced aging recall these pathologies and may represent the consequences of prooxidant and proinflammatory conditions. The role of inflammatory cytokines released by activated microglia and astrocytes in synaptic plasticity and its age-related modulation has been shown for major hippocampal synaptic systems (see for recent reviews [74, 75]), whereas cytokine contribution to age-related alterations in corticostriatal plasticity remains to be investigated.

## 5. Conclusion

Comparative analysis of age-dependent alterations in several forms of corticostriatal plasticity and striatal gene expression in mice showed that age-related alterations in plasticity may be associated with functional changes in receptor-activated signaling cascades rather than with receptor gene expression. These functional changes may result from the gradually increasing neuroinflammation and the concomitant oxidative stress.

## Supplementary Material

Supplementary material provides a list of genes (with reference to the Gene Bank sequences) analysed with the PCR array PAMM-062Z (SA Biosciences) in Table 1 as well as sequences and features of the primers used for the conventional semiquantitative real-time PCR performed for the validation of gene array data (Table 2). Linear regression coefficients and efficiency of the amplification provided in Table 2 for each primer pair were determined from the standard curves and illustrate suitability of selected primer pairs for the gene expression quantification.

## Figures and Tables

**Table 1:** Expression levels of indicated genes involved in synaptic plasticity, oxidative stress, and immunity. mRNA levels are normalized to group I values.

Relative mRNA level %	Age groups		
	II	III	IV
Synaptic plasticity			
NR1 (Grin1)	35 ± 8 (7)^***^	28 ± 5 (4)^***^	68 ± 3 (4)^*^
NR 2A	44 ± 5 (4)^*^	60 ± 13 (5)	73 ± 9 (7)
NR 2B	61 ± 8 (6)^*^	96 ± 17 (6)	79 ± 5 (9)
NR 2C	75 ± 12 (4)	56 ± 7 (4)	70 ± 10 (8)
mGluR 1	98 ± 8 (4)	100 ± 15 (4)	88 ± 11 (6)
mGluR 5	106 ± 8 (4)	113 ± 9 (4)	81 ± 16 (6)
D1R	81 ± 12 (4)	88 ± 19 (4)	44 ± 16 (4)^*^
D2R	91 ± 11 (6)	130 ± 28 (5)	83 ± 11 (8)
CB1R	106 ± 23 (4)	96 ± 25 (5)	94 ± 15 (8)
nNOS	75 ± 16 (5)	66 ± 6 (6)^*^	75 ± 6 (7)^*^
eNOS	131 ± 30 (4)	113 ± 20 (3)	137 ± 16 (7)
Oxidative stress			
Txnip	72 ± 15 (4)	58 ± 11 (5)	118 ± 28 (7)
Cyba	80 ± 9 (4)	69 ± 10 (5)	195 ± 35 (7)^*^
Gpx6	89 ± 15 (6)	142 ± 24 (6)	143 ± 17 (8)^*^
Scd2	113 ± 27 (4)	116 ± 21 (5)	141 ± 18 (9)^*^
Immunity			
B2m	144 ± 12 (4)	221 ± 63 (5)^*^	230 ± 30 (9)^**^
Lcn2	122 ± 41 (5)	215 ± 68 (6)^*^	474 ± 126 (7)^**^
Irgm1	152 ± 20 (4)	165 ± 23 (5)	229 ± 31 (9)^***^

Significant differences with this group are indicated by asterisks: ^*^ *P* < 0.05, ^**^ *P* < 0.01, ^***^ *P* < 0.001, one-way ANOVA with Dunnett's posttest and Mann-Whitney *U* test.

**Table 2:** The magnitude and occurrence of changes in corticostriatal field responses induced by high-frequency stimulation (HFS) in striatal slices from the brain of four age groups of mice. The magnitude of LTD/LTP was measured as the mean relative change in field response amplitude within 45–60 min after HFS.

Post-HFS changes in responses	Age groups			
	I	II	III	IV
Long-term depression, LTD	71 ± 2% *n* = 8 (36%)	48 ± 7%^***^ *n* = 5 (38%)	71 ± 1% *n* = 2 (18%)	62 ± 5% *n* = 8 (30%)

Long-term potentiation, LTP	126 ± 2% *n* = 8 (36%)	171 ± 9%^***^ *n* = 7 (54%)	165 ± 9%^***^ *n* = 6 (55%)	136 ± 2%^**^ *n* = 13 (48%)

No long-term changes NLTC	101 ± 2% *n* = 6 (28%)	100 ± 2% *n* = 1 (8%)	94 ± 1% *n* = 3 (27%)	97 ± 2% *n* = 6 (22%)

*n* indicates the number of slices with its percent ratio to the total number of slices in a group in parentheses. Asterisks indicate significant difference in the magnitude of LTD/LTP with respect to group I values (^**^ *P* < 0.01, ^***^ *P* < 0.001, one-way ANOVA with Dunnett's posttest and unpaired *t*-test).

## References

[1] Kalpouzos G., Persson J., Nyberg L. (2012). Local brain atrophy accounts for functional activity differences in normal aging. *Neurobiology of Aging*.

[2] Langenecker S. A., Briceno E. M., Hamid N. M., Nielson K. A. (2007). An evaluation of distinct volumetric and functional MRI contributions toward understanding age and task performance: a study in the basal ganglia. *Brain Research*.

[3] Marchand W. R., Lee J. N., Suchy Y. (2011). Age-related changes of the functional architecture of the cortico-basal ganglia circuitry during motor task execution. *NeuroImage*.

[4] Calabresi P., Centonze D., Gubellini P. (2000). Synaptic transmission in the striatum: from plasticity to neurodegeneration. *Progress in Neurobiology*.

[5] Kreitzer A. C., Malenka R. C. (2008). Striatal plasticity and basal ganglia circuit function. *Neuron*.

[6] Mahon S., Deniau J.-M., Charpier S. (2004). Corticostriatal plasticity: life after the depression. *Trends in Neurosciences*.

[7] Centonze D., Picconi B., Gubellini P., Bernardi G., Calabresi P. (2001). Dopaminergic control of synaptic plasticity in the dorsal striatum. *European Journal of Neuroscience*.

[8] Centonze D., Gubellini P., Pisani A., Bernardi G., Calabresi P. (2003). Dopamine, acetylcholine, and nitric oxide systems interact to induce corticostriatal synaptic plasticity. *Reviews in the Neurosciences*.

[9] Darbin O. (2012). The aging striatal dopamine function. *Parkinsonism and Related Disorders*.

[10] Mora F., Segovia G., del Arco A. (2008). Glutamate-dopamine-GABA interactions in the aging basal ganglia. *Brain Research Reviews*.

[11] Ossowska K. (1993). Disturbances in neurotransmission processes in aging and age-related diseases. *Polish Journal of Pharmacology*.

[12] Sherman K. A., Friedman E. (1990). Pre- and post-synaptic cholinergic dysfunction in aged rodent brain regions: new findings and an interpretative review. *International Journal of Developmental Neuroscience*.

[13] Zoli M., Ferraguti F., Toffano G., Fuxe K., Agnati L. F. (1993). Neurochemical alterations but not nerve cell loss in aged rat neostriatum. *Journal of Chemical Neuroanatomy*.

[14] Akopian G., Walsh J. P. (2006). Pre- and postsynaptic contributions to age-related alterations in corticostriatal synaptic plasticity. *Synapse*.

[15] Wang Y. (2008). Differential effect of aging on synaptic plasticity in the ventral and dorsal striatum. *Neurobiology of Learning and Memory*.

[16] West A. R., Tseng K. Y. (2011). Nitric oxide-soluble guanylyl cyclase-cyclic GMP signaling in the striatum: new targets for the treatment of Parkinson's disease?. *Frontiers in Systems Neuroscience*.

[17] Calabresi P., Gubellini P., Centonze D. (1999). A critical role of the nitric oxide/cGMP pathway in corticostriatal long-term depression. *Journal of Neuroscience*.

[18] Doreulee N., Sergeeva O. A., Yanovsky Y. (2003). Cortico-striatal synaptic plasticity in endothelial nitric oxide synthase deficient mice. *Brain Research*.

[19] Sammut S., Threlfell S., West A. R. (2010). Nitric oxide-soluble guanylyl cyclase signaling regulates corticostriatal transmission and short-term synaptic plasticity of striatal projection neurons recorded in vivo. *Neuropharmacology*.

[20] Sergeeva O. A., Doreulee N., Chepkova A. N., Kazmierczak T., Haas H. L. (2007). Long-term depression of cortico-striatal synaptic transmission by DHPG depends on endocannabinoid release and nitric oxide synthesis. *European Journal of Neuroscience*.

[21] Cha C. I., Sohn S. G., Chung Y. H., Shin C.-M., Baik S. H. (2000). Region-specific changes of NOS-IR cells in the basal ganglia of the aged rat. *Brain Research*.

[22] Yamada K., Noda Y., Komori Y., Sugihara H., Hasegawa T., Nabeshima T. (1996). Reduction in the number of NADPH-diaphorase-positive cells in the cerebral cortex and striatum in aged rats. *Neuroscience Research*.

[23] Domek-Łopacińska K., van de Waarenburg M., van Ittersum M. M., Steinbusch H. W. M., de Vente J. (2005). Nitric oxide-induced cGMP synthesis in the cholinergic system during the development and aging of the rat brain. *Developmental Brain Research*.

[24] Necchi D., Virgili M., Monti B., Contestabile A., Scherini E. (2002). Regional alterations of the NO/NOS system in the aging brain: a biochemical, histochemical and immunochemical study in the rat. *Brain Research*.

[25] Pérez-Severiano F., Escalante B., Vergara P., Ríos C., Segovia J. (2002). Age-dependent changes in nitric oxide synthase activity and protein expression in striata of mice transgenic for the Huntington's disease mutation. *Brain Research*.

[26] Zhuo L., Sun B., Zhang C.-L., Fine A., Chiu S.-Y., Messing A. (1997). Live astrocytes visualized by green fluorescent protein in transgenic mice. *Developmental Biology*.

[27] Yanovsky Y., Li S., Klyuch B. P. (2011). L-Dopa activates histaminergic neurons. *The Journal of Physiology*.

[28] Schmittgen T. D., Livak K. J. (2008). Analyzing real-time PCR data by the comparative CT method. *Nature Protocols*.

[29] Tanaka C., Coling D. E., Manohar S. (2012). Expression pattern of oxidative stress and antioxidant defense-related genes in the aging Fischer 344/NHsd rat cochlea. *Neurobiology of Aging*.

[30] Pfaffl M. W. (2001). A new mathematical model for relative quantification in real-time RT-PCR. *Nucleic Acids Research*.

[31] Cevenini E., Caruso C., Candore G. (2010). Age-related inflammation: the contribution of different organs, tissues and systems. How to face it for therapeutic approaches. *Current Pharmaceutical Design*.

[32] Linnemann D., Skarsfelt T. (1994). Regional changes in expression of NCAM, GFAP, and S100 in aging rat brain. *Neurobiology of Aging*.

[33] Nichols N. R., Day J. R., Laping N. J., Johnson S. A., Finch C. E. (1993). GFAP mRNA increases with age in rat and human brain. *Neurobiology of Aging*.

[34] Yuan R., Tsaih S.-W., Petkova S. B. (2009). Aging in inbred strains of mice: study design and interim report on median lifespans and circulating IGF1 levels. *Aging Cell*.

[35] Malenka R. C., Kocsis J. D. (1988). Presynaptic actions of carbachol and adenosine on corticostriatal synaptic transmission studied in vitro. *The Journal of Neuroscience*.

[36] Mitchell J. J., Anderson K. J. (1998). Age-related changes in [3H]MK-801 binding in the Fischer 344 rat brain. *Neurobiology of Aging*.

[37] Picconi B., Bagetta V., Ghiglieri V. (2011). Inhibition of phosphodiesterases rescues striatal long-term depression and reduces levodopa-induced dyskinesia. *Brain*.

[38] Tozzi A., Costa C., Siliquini S. (2012). Mechanisms underlying altered striatal synaptic plasticity in old A53T-*α* synuclein overexpressing mice. *Neurobiology of Aging*.

[39] Chepkova A. N., Selbach O., Haas H. L., Sergeeva O. A. (2012). Ammonia-induced deficit in corticostriatal long-term depression and its amelioration by zaprinast. *Journal of Neurochemistry*.

[40] Adermark L., Lovinger D. M. (2007). Retrograde endocannabinoid signaling at striatal synapses requires a regulated postsynaptic release step. *Proceedings of the National Academy of Sciences of the United States of America*.

[41] Gerdeman G. L., Ronesi J., Lovinger D. M. (2002). Postsynaptic endocannabinoid release is critical to long-term depression in the striatum. *Nature Neuroscience*.

[42] Gubellini P., Saulle E., Centonze D. (2001). Selective involvement of mGlu1 receptors in corticostriatal LTD. *Neuropharmacology*.

[43] Calabresi P., Gubellini P., Centonze D. (2000). Dopamine and cAMP-regulated phosphoprotein 32 kDa controls both striatal long-term depression and long-term potentiation, opposing forms of synaptic plasticity. *The Journal of Neuroscience*.

[44] Nishi A., Watanabe Y., Higashi H., Tanaka M., Nairn A. C., Greengard P. (2005). Glutamate regulation of DARPP-32 phosphorylation neostriatal neurons involves activation of multiple signaling cascades. *Proceedings of the National Academy of Sciences of the United States of America*.

[45] Svenningsson P., Nishi A., Fisone G., Girault J.-A., Nairn A. C., Greengard P. (2004). DARPP-32: an integrator of neurotransmission. *Annual Review of Pharmacology and Toxicology*.

[46] Magnone M. C., Rossolini G., Piantanelli L., Migani P. (2000). Neurochemical parameters of the main neurotransmission systems in aging mice. *Archives of Gerontology and Geriatrics*.

[47] Magnusson K. R., Cotman C. W. (1993). Age-related changes in excitatory amino acid receptor in two mouse strains. *Neurobiology of Aging*.

[48] Wardas J., Pietraszek M., Schulze G., Ossowska K., Wolfarth S. (1997). Age-related changes in glutamate receptors: an autoradiographic analysis. *Polish Journal of Pharmacology*.

[49] Cepeda C., Li Z., Levine M. S. (1996). Aging reduces neostriatal responsiveness to N-methyl-D-aspartate and dopamine: an in vitro electrophysiological study. *Neuroscience*.

[50] Flores-Hernández J., Cepeda C., Hernández-Echeagaray E. (2002). Dopamine enhancement of NMDA currents in dissociated medium-sized striatal neurons: role of D1 receptors and DARPP-32. *Journal of Neurophysiology*.

[51] Kheirbek M. A., Britt J. P., Beeler J. A., Ishikawa Y., McGehee D. S., Zhuang X. (2009). Adenylyl cyclase type 5 contributes to corticostriatal plasticity and striatum-dependent learning. *The Journal of Neuroscience*.

[52] Giorgi O., Calderini G., Toffano G., Biggio G. (1987). D-1 dopamine receptors labelled with 3H-SCH 23390: decrease in the striatum of aged rats. *Neurobiology of Aging*.

[53] Mancini M., Ricci A., Zaccheo D., Amenta F. (1991). Similarity of age-dependent changes in renal and striatal dopamine receptors. *Functional Neurology*.

[54] May T., Sugawa M. (1993). Altered dopamine receptor mediated signal transmission in the striatum of aged rats. *Brain Research*.

[55] Suzuki M., Hatano K., Sakiyama Y., Kawasumi Y., Kato T., Ito K. (2001). Age-related changes of dopamine D1-like and D2-like receptor binding in the F344/N rat striatum revealed by positron emission tomography and in vitro receptor autoradiography. *Synapse*.

[56] Floyd R. A., Hensley K. (2002). Oxidative stress in brain aging: implications for therapeutics of neurodegenerative diseases. *Neurobiology of Aging*.

[57] Mariani E., Polidori M. C., Cherubini A., Mecocci P. (2005). Oxidative stress in brain aging, neurodegenerative and vascular diseases: an overview. *Journal of Chromatography B: Analytical Technologies in the Biomedical and Life Sciences*.

[58] Yap L. P., Garcia J. V., Han D., Cadenas E. (2009). The energy-redox axis in aging and age-related neurodegeneration. *Advanced Drug Delivery Reviews*.

[59] Arranz L., Naudí A., de la Fuente M., Pamplona R. (2013). Exceptionally old mice are highly resistant to lipoxidation-derived molecular damage. *Age (Dordr)*.

[60] Choi D.-Y., Zhang J., Bing G. (2010). Aging enhances the neuroinflammatory response and *α*-synuclein nitration in rats. *Neurobiology of Aging*.

[61] Tadros S. F., D'Souza M., Zhu X., Frisina R. D. (2014). Gene expression changes for antioxidants pathways in the mouse cochlea: relations to age-related hearing deficits. *PLoS ONE*.

[62] Villar-Cheda B., Valenzuela R., Rodriguez-Perez A. I., Guerra M. J., Labandeira-Garcia J. L. (2012). Aging-related changes in the nigral angiotensin system enhances proinflammatory and pro-oxidative markers and 6-OHDA-induced dopaminergic degeneration. *Neurobiology of Aging*.

[63] Babior B. M. (2004). NADPH oxidase. *Current Opinion in Immunology*.

[64] Cai H. (2005). NAD(P)H oxidase-dependent self-propagation of hydrogen peroxide and vascular disease. *Circulation Research*.

[65] Dikalov S. (2011). Cross talk between mitochondria and NADPH oxidases. *Free Radical Biology and Medicine*.

[66] Gorin Y., Block K. (2013). Nox as a target for diabetic complications. *Clinical Science*.

[67] Lassègue B., San Martín A., Griendling K. K. (2012). Biochemistry, physiology, and pathophysiology of NADPH oxidases in the cardiovascular system. *Circulation Research*.

[68] Morré D. J., Morré D. M. (2003). Cell surface NADH oxidases (ECTO-NOX proteins) with roles in cancer, cellular time-keeping, growth, aging and neurodegenerative diseases. *Free Radical Research*.

[69] Wilkinson B. L., Landreth G. E. (2006). The microglial NADPH oxidase complex as a source of oxidative stress in Alzheimer's disease. *Journal of Neuroinflammation*.

[70] Jha M. K., Lee S., Park D. H. (2015). Diverse functional roles of lipocalin-2 in the central nervous system. *Neuroscience & Biobehavioral Reviews*.

[71] Akopian G., Crawford C., Petzinger G., Jakowec M. W., Walsh J. P. (2012). Brief mitochondrial inhibition causes lasting changes in motor behavior and corticostriatal synaptic physiology in the Fischer 344 rat. *Neuroscience*.

[72] Belujon P., Lodge D. J., Grace A. A. (2010). Aberrant striatal plasticity is specifically associated with dyskinesia following levodopa treatment. *Movement Disorders*.

[73] Dalbem A., Silveira C. V., Pedroso M. F. (2005). Altered distribution of striatal activity-dependent synaptic plasticity in the 3-nitropropionic acid model of Huntington's disease. *Brain Research*.

[74] Lynch M. A. (2010). Age-related neuroinflammatory changes negatively impact on neuronal function. *Frontiers in Aging Neuroscience*.

[75] Yirmiya R., Goshen I. (2011). Immune modulation of learning, memory, neural plasticity and neurogenesis. *Brain, Behavior, and Immunity*.

