# Supplementary material for: Age-Related Alterations in the Expression of Genes and Synaptic Plasticity Associated with Nitric Oxide Signaling in the Mouse Dorsal Striatum
Source: Neural Plast. 2015 Mar 4;2015:458123. doi: 10.1155/2015/458123 (PMC4364378; doi:10.1155/2015/458123)
Supplement: Supplementary file 1 — Supplementary material provides a list of genes (with reference to the Gene Bank sequences) analysed with the PCR array PAMM-062Z (SA Biosciences) in Table 1 as well as sequences and features of the primers used for the conventional semiquantitative real-time PCR performed for the validation of gene array data (Table 2). Linear regression coefficients and efficiency of the amplification provided in Table 2 for each primer pair were determined from the standard curves and illustrate suitability of selected primer pairs for the gene expression quantification. [file 458123.f1.pdf]

Supplementary Table 1

PAMM-062Z

Position	Refseq	Symbol	Description
A01	NM_013930	Aass	Aminoadipate-semialdehyde synthase
A02	NM_028717	Als2	Amyotrophic lateral sclerosis 2 (juvenile) homolog (human)
A03	NM_009696	Apoe	Apolipoprotein E
A04	NM_007527	Bax	Bcl2-associated X protein
A05	NM_009743	Bcl2l1	Bcl2-like 1
A06	NM_009790	Calm1	Calmodulin 1
A07	NM_009795	Capns1	Calpain, small subunit 1
A08	NM_009804	Cat	Catalase
A09	NM_007616	Cav1	Caveolin 1, caveolae protein
A10	NM_007631	Ccnd1	Cyclin D1
A11	NM_009831	Ccng1	Cyclin G1
A12	NM_016892	Ccs	Copper chaperone for superoxide dismutase
B01	NM_009842	Cd151	CD151 antigen
B02	NM_007669	Cdkn1a	Cyclin-dependent kinase inhibitor 1A (P21)
B03	NM_019952	Clcf1	Cardiotrophin-like cytokine factor 1
B04	NM_007798	Ctsb	Cathepsin B
B05	NM_007806	Cyba	Cytochrome b-245, alpha polypeptide
B06	NM_026993	Ddah1	Dimethylarginine dimethylaminohydrolase 1
B07	NM_016765	Ddah2	Dimethylarginine dimethylaminohydrolase 2
B08	NM_007864	Dlg4	Discs, large homolog 4 (Drosophila)
B09	NM_019682	Dynll1	Dynein light chain LC8-type 1
B10	NM_007913	Egr1	Early growth response 1
B11	NM_007946	Epx	Eosinophil peroxidase
B12	NM_007949	Ercc2	Excision repair cross-complementing rodent repair deficiency, complementation group 2
C01	NM_007987	Fas	Fas (TNF receptor superfamily member 6)
C02	NM_010234	Fos	FBJ osteosarcoma oncogene
C03	NM_021356	Gab1	Growth factor receptor bound protein 2-associated protein 1
C04	NM_007836	Gadd45a	Growth arrest and DNA-damage-inducible 45 alpha
C05	NM_008160	Gpx1	Glutathione peroxidase 1
C06	NM_030677	Gpx2	Glutathione peroxidase 2
C07	NM_008161	Gpx3	Glutathione peroxidase 3
C08	NM_008162	Gpx4	Glutathione peroxidase 4
C09	NM_010343	Gpx5	Glutathione peroxidase 5
C10	NM_145451	Gpx6	Glutathione peroxidase 6
C11	NM_024198	Gpx7	Glutathione peroxidase 7
C12	NM_008169	Grin1	Glutamate receptor, ionotropic, NMDA1 (zeta 1)
D01	NM_010439	Hmgb1	High mobility group box 1
D02	NM_008281	Hpn	Hepsin
D03	NM_010497	Idh1	Isocitrate dehydrogenase 1 (NADP+), soluble
D04	NM_008326	Irgm1	Immunity-related GTPase family M member 1
D05	NM_010786	Mdm2	Transformed mouse 3T3 cell double minute 2
D06	NM_026119	Med4	Mediator of RNA polymerase II transcription, subunit 4 homolog (yeast)
D07	NM_010824	Mpo	Myeloperoxidase
D08	NM_010849	Myc	Myelocytomatosis oncogene
D09	NM_010877	Ncf2	Neutrophil cytosolic factor 2
D10	NM_008682	Nedd1	Neural precursor cell expressed, developmentally down-regulated gene 1
D11	NM_008712	Nos1	Nitric oxide synthase 1, neuronal
D12	NM_010927	Nos2	Nitric oxide synthase 2, inducible
E01	NM_008713	Nos3	Nitric oxide synthase 3, endothelial cell

E02	NM_172203	Nox1	NADPH oxidase 1
E03	NM_015760	Nox4	NADPH oxidase 4
E04	NM_172204	Noxa1	NADPH oxidase activator 1
E05	NM_027988	Noxo1	NADPH oxidase organizer 1
E06	NM_172527	Nudt15	Nudix (nucleoside diphosphate linked moiety X)-type motif 15
E07	NM_011063	Pea15a	Phosphoprotein enriched in astrocytes 15A
E08	NM_133819	Ppp1r15b	Protein phosphatase 1, regulatory (inhibitor) subunit 15b
E09	NM_008913	Ppp3ca	Protein phosphatase 3, catalytic subunit, alpha isoform
E10	NM_011034	Prdx1	Peroxiredoxin 1
E11	NM_011563	Prdx2	Peroxiredoxin 2
E12	NM_007453	Prdx6	Peroxiredoxin 6
F01	NM_008854	Prkaca	Protein kinase, cAMP dependent, catalytic, alpha
F02	NM_011170	Prnp	Prion protein
F03	NM_027297	Prpf4	PRP4 pre-mRNA processing factor 4 homolog (yeast)
F04	NM_011186	Psmb5	Proteasome (prosome, macropain) subunit, beta type 5
F05	NM_008987	Ptx3	Pentraxin related gene
F06	NM_009029	Rb1	Retinoblastoma 1
F07	NM_058214	Recql4	RecQ protein-like 4
F08	NM_023396	Rprm	Reprimo, TP53 dependent G2 arrest mediator candidate
F09	NM_009101	Rras	Harvey rat sarcoma oncogene, subgroup R
F10	NM_010925	Rrp1	Ribosomal RNA processing 1 homolog (S. cerevisiae)
F11	NM_009127	Scd1	Stearoyl-Coenzyme A desaturase 1
F12	NM_009128	Scd2	Stearoyl-Coenzyme A desaturase 2
G01	NM_024450	Scd3	Stearoyl-coenzyme A desaturase 3
G02	NM_018754	Sfn	Stratifin
G03	NM_011434	Sod1	Superoxide dismutase 1, soluble
G04	NM_013671	Sod2	Superoxide dismutase 2, mitochondrial
G05	NM_011435	Sod3	Superoxide dismutase 3, extracellular
G06	NM_009417	Tpo	Thyroid peroxidase
G07	NM_011640	Trp53	Transformation related protein 53
G08	NM_023719	Txnip	Thioredoxin interacting protein
G09	NM_013711	Txnrd2	Thioredoxin reductase 2
G10	NM_019639	Ubc	Ubiquitin C
G11	NM_009464	Ucp3	Uncoupling protein 3 (mitochondrial, proton carrier)
G12	NM_011728	Xpa	Xeroderma pigmentosum, complementation group A
H01	NM_007393	Actb	Actin, beta
H02	NM_009735	B2m	Beta-2 microglobulin
H03	NM_008084	Gapdh	Glyceraldehyde-3-phosphate dehydrogenase
H04	NM_010368	Gusb	Glucuronidase, beta
H05	NM_008302	Hsp90ab1	Heat shock protein 90 alpha (cytosolic), class B member 1
H06	SA_00106	MGDC	Mouse Genomic DNA Contamination
H07	SA_00104	RTC	Reverse Transcription Control
H08	SA_00104	RTC	Reverse Transcription Control
H09	SA_00104	RTC	Reverse Transcription Control
H10	SA_00103	PPC	Positive PCR Control
H11	SA_00103	PPC	Positive PCR Control
H12	SA_00103	PPC	Positive PCR Control

Supplementry Table 1. Complete gene list of gene array PAMM-062Z.

Supplementary Table 2

Gene	GeneBank #	sense primer	antisense primer	size,bp	r²	E
Aass	NM_013930	CTCTGGCTCCCAAGCACATC	CCCAAAATCAGACAGGCTTCT	151	0,98	1,8
Aif1 / IBA1	NM_0194672	GTCCTTGAAGCGAATGCTGG	CATTCTCAAGATGGCAGATC	157	0,96	2,4
Als2	NM_028717	GAGAGCTTTTGCTAATGGGATGG	GCCTGGCAACCTCTCTGGT	162	0,95	2,1
B2m	NM_009735	ACTGACCGGCCTGTATGCTA	CAGTCTCAGTGGGGGTGAAT	244	0,99	2,2
CB1R	NM_007726	GTGGGCAGCCTGTTCCTCA	CATGCGGGCTTGGTCAGG	401	0,99	1,9
Cyba	NM_007806	CATCGTGGCTACTGCTGGAC	TGGACCCCTTTTTCCTCTTT	116	0,96	2,2
D1R/Drd1a	XM_006516848	CAGAACTGTGACACAAGGTTGAGC	CTGGCAATTCTTGGCATGGACTG	177	0,99	1,6
D2R	NM_010077	GCAGTCCGAGCTTTCAGAGCC	GCTATACCGGGTCCTCTCTGG	206	0,98	1,9
GapdH	NM_001001303	TGTGTCCGTCGTGGATCTGA	TTGCTGTTGAACTCGCAGGAG	150	0,99	1,9
GFAP	XR_388347	ACCAGCTTACGGCCAACAGTG	TGTCTATACGCAGCCAGGTTGTTC	138	0,995	2,14
GLT-1	KC825358	CTCACTGACTGTGTTTGGTG	GAGGTGCCACCAGAACTTTC	457	0,99	2
Glul /GS	NM_008131	CACTACCGGGCCTGCTTGTAT	GCATGGCCTTGGTGCTAAAGTTG	249	0,997	2,1
GluN1/NR1/Grin1	NM_008169	GCTGTACCTGCTGGACCGCT	GCAGTGAGGAAGCCACTATGATC	218	0,99	1,9
GluN2A (NR2A)	NM_008170	GGTTCTGCATCGACATCC	GTGAGCCCACAGCCATGACT	162	0,99	1,8
GluN2B /NR2B	NM_008171	GCTACAACACCCACGAGAAGAG	GAGAGGGTCCACGCTTTCC	314	0,97	2,5
GluN2C (NR2C)	NM_010350	GCTTCTGCATCGACATCC	CCATGTCTGCCCGCTTGTA	150	0,98	1,8
Gpx6	NM_145451	GCCCAGAAGTTGTGGGGTTC	TCCATACTCATAGACGGTGCC	129	0,98	2,2
Hsp90	NM_008302	GTGCGAGTCGGACTTGGTCCG	TGCCTGAAAGGCAAAGGTCTCC	123	0,99	1,8
Irgm1	NM_008326	ACGAAGACGCGGACTGAGTACT	CAGAGGCAATGATGAATAAGTCAC	82	0,99	2,2
Lcn2	NM_008491	CCAGTTCGCCATGGTATTT	GGGTGAAACGTTCCTTCAGT	113	0,98	2,3
MAG	NM_010758	GAGACATCCCCAACTCAGGGAGAC	TGTGCGGGACTTGAAGACCA	290	0,99	1,9
mGluR1	NM_016976	CCATTTTGTCCTACCCG	GTCATGATCTTGTAGTTGCT	246	0,97	2
mGluR5	NM_001081414	GGATATAATGCATGACTATCCA	GTCATGATTTTGTAGTTGCT	232	0,98	2
eNOS	NM_008713	GCAAGACAGACTACACGACATTGA	GAGTACCTCGTCCCGGTAGA	112	0,94	2,1
nNOS	NM_008712	AGTTTTTGGCAACAGCGACA	GCAGGGTCTCCTCTCTGTAGATATG	121	0,99	1,9
Pea15a	NM_011063	CAAGGAGGACATCCCCAGT	CTTCAGCACACGGGTTCTGTA	184	0,98	2
Scd1	NM_009127	TCTTGTCCCTATAGCCCAATCCAG	AGCTCAGAGCGCGTGTTCAA	130	0,98	2,3
Scd2	NM_009128	AGTGTTGCTCGTGAGCCTGTG	CCTGCAGATCCATGTCCAGCTA	140	0,99	1,9
Scd3	NM_024450	TCACACCGTGAACCCTGAGATTGT	TGCTTGCTCTGCCTCTTGACCTAT	160	0,99	2
S100ß	NM_009115	ACGGCTTCCTGCTGGTGTTTG	GAGAGAGCTCGTTGTTGATAAGCT	101	0,93	1,9
Txnip	NM_023719	CAAGTTCGGCTTCGAGCTTC	ACACTTACCTTGCCAATGGC	379	0,98	1,9

Supplementary Table2. Primers for real-time RT-PCR. Genebank #: shows reference sequence from NCBI, to which sequenced PCR products had highest homology. Aif1/IBA1, a macrophage/microglia-specific calcium-binding protein; GLT-1: glutamate transporter 1; Glul: glutamate ligase, also known as glutamine synthetase (GS); Lcn2: lipocalin-2, also called oncogene 24p3; MAG: oligodendrocyte marker myelin-associated glycoprotein. For the description of other Gene abbreviations see Supplementary Table1 or text of the manuscript.
